# Hypogonadism and sexual function in men affected by adrenocortical carcinoma under mitotane therapy

**DOI:** 10.3389/fendo.2023.1320722

**Published:** 2024-01-10

**Authors:** Letizia Canu, Clotilde Sparano, Lara Naletto, Giuseppina De Filpo, Giulia Cantini, Elena Rapizzi, Serena Martinelli, Tonino Ercolino, Francesca Cioppi, Alessandro Fantoni, Lorenzo Zanatta, Alessandro Terreni, Massimo Mannelli, Michaela Luconi, Mario Maggi, Francesco Lotti

**Affiliations:** ^1^ Endocrinology Unit, Department of Experimental and Clinical Biomedical Sciences “Mario Serio”, University of Florence, Florence, Italy; ^2^ Endocrinology Unit, Careggi University Hospital (AOUC), Florence, Italy; ^3^ Centro di Ricerca e Innovazione sulle Patologie Surrenaliche, AOU Careggi, Florence, Italy; ^4^ Center of Excellence of European Network for the Study of Adrenal Tumors (ENS@T), Florence, Italy; ^5^ Department of Experimental and Clinical Medicine, University of Florence, Florence, Italy; ^6^ Department of Laboratory, Careggi University Hospital (AOUC), Florence, Italy; ^7^ Andrology, Female Endocrinology and Gender Incongruence Unit, Department of Experimental and Clinical Biomedical Sciences “Mario Serio”, University of Florence, Florence, Italy

**Keywords:** adrenocortical carcinoma, mitotane, hypergonadotropic hypogonadism, androgen replacement therapy, sexual dysfunction

## Abstract

**Purpose:**

Adrenocortical carcinoma (ACC) is a rare and aggressive tumor. ACC male patients under adjuvant mitotane therapy (AMT) frequently develop hypogonadism, however sexual function has never been assessed in this setting. The aim of this retrospective study was to evaluate in AMT treated ACC patients the changes in Luteinizing hormone (LH), Sex Hormone Binding Globulin (SHBG), total testosterone (TT) and calculated free testosterone (cFT), the prevalence and type of hypogonadism and sexual function, the latter before and after androgen replacement therapy (ART).

**Methods:**

LH, SHBG, TT and cFT were assessed in ten ACC patients at baseline (T0) and six (T1), twelve (T2), and eighteen (T3) months after AMT. At T3, ART was initiated in eight hypogonadal patients, and LH, SHBG, TT and cFT levels were evaluated after six months (T4). In six patients, sexual function was evaluated before (T3) and after (T4) ART using the International Index of Erectile Function-15 (IIEF-15) questionnaire.

**Results:**

Under AMT we observed higher SHBG and LH and lower cFT levels at T1-T3 compared to T0 (all p<0.05). At T3, hypergonadotropic hypogonadism and erectile dysfunction (ED) were detected in 80% and 83.3% of cases. At T4, we observed a significant cFT increase in men treated with T gel, and a significant improvement in IIEF-15 total and subdomains scores and ED prevalence (16.7%) in men under ART.

**Conclusion:**

AMT was associated with hypergonatropic hypogonadism and ED, while ART led to a significant improvement of cFT levels and sexual function in the hypogonadal ACC patients. Therefore, we suggest to evaluate LH, SHBG, TT and cFT and sexual function during AMT, and start ART in the hypogonadal ACC patients with sexual dysfunction.

## Introduction

Adrenocortical carcinoma (ACC) is a rare tumor (0.7–2.0 cases per million persons/year) (1, 2), with a slight female preponderance (M:F 1.0:1.2) (3). The relative peak of incidence is in the fourth and fifth decades of life (4). ACC is associated with a poor prognosis and with an average 5-years survival rate of 50%, which dramatically drops to 6.9 – 13.0% in patients with documented distant metastases (5, 6). Currently, surgery represents the only curative approach for ACC patients (7). However, the complete tumor resection is often not curative due to the high risk of recurrence (8). For this reason, international guidelines recommend adjuvant therapies after surgery (9).

In particular, the latest guidelines of the European Network for the Study of Adrenal Tumors (ENSAT) recommend mitotane as an adjuvant therapy for at least two years after surgery in patients with a high risk of recurrence (ENSAT stage III, or R1 resection, or Ki67 > 10%) (9). Mitotane is a synthetic derivative of dichloro-diphenyl-trichloroethane (DDT) with a direct cytotoxic effect on the adrenal cortex (10–12), and has been approved by the European Medicines Agency (EMA) as therapy for ACC patients with advanced inoperable, or metastatic, or cortisol secreting masses. Recent evidences confirmed that adjuvant mitotane therapy (AMT) reduces the risk of recurrence and death (13). In addition, a high mitotane “time in target range” (TTR, months with mitotane levels 14-20 mg/l) was associated with a reduced risk of ACC recurrence in an adjuvant setting (14) and of death in a palliative group (15).

Nevertheless, mitotane has several side effects that are responsible of a limited tolerability (16). In males, one of the most frequent adverse effects is hypogonadism, which has been reported in 35.6% of men (17). The main reason suggested for AMT-related hypogonadism is that mitotane causes a rise in the hepatic SHBG synthesis and release (18) which can lead to a reduction in cFT levels. Despite this evidence, the hormonal status in ACC patients under AMT has been evaluated only in four studies (19–22), with partially discordant results, which might in part be due to differences in mitotane regimen, timing of blood sampling and laboratory methods for mitotane measurement, as well as in the circulating levels of the drug.

On the other hand, it is well known that hypogonadism, as well as malignancies *per se*, are associated with sexual dysfunction (23). However, no study evaluated the sexual function and the impact of androgen replacement therapy (ART) on sexuality in surgically treated ACC men under AMT.

The aim of the present study was to evaluate in surgically treated ACC patients under AMT: (i) the changes in LH, SHBG, TT, and cFT levels and the prevalence and type of hypogonadism during AMT, as well as (ii), for the first time, the sexual function before and after ART in patients with hypogonadism.

## Materials and methods

### Patients

We studied retrospectively ten surgically treated ACC male patients attending the Endocrinology Unit of the University Hospital of Florence. In particular, we included in this study a consecutive series of patients affected by histologically confirmed ACC treated with AMT between January 1^st^, 2013 and December 31, 2020. A comprehensive anamnesis (including age, smoking habit and alcohol consumption), as well as a general (body mass index, waistline, systolic and diastolic arterial blood pressure) and andrological (including testis volume and male breast assessment) physical examination were performed according to a previous study (24).

AMT was initiated after radical surgery with the intention of at least two years of therapy, although an individual approach was adopted considering the patients’ compliance and the side effect tolerability (9). In particular, AMT was started with a minimum daily dose of 1000 mg and was increased up to the maximally tolerated dose. We evaluated the minimum and maximum daily dose of mitotane, the mean and maximum levels reached and the “time in target range” (TTR) corresponding to the number of months in which mitotane concentrations were between 14 and 20 mg/l (15). The mitotane levels were evaluated every month until the achievement of 14 mg/l and then every two-three months. We also registered the start date and type of any supportive/replacement therapy (glucocorticoid, fludrocortisone, levothyroxine, lipid lowering, ART) initiated due to ACC or ACC surgery-related hormonal deficiencies/metabolic abnormalities. In particular, ART was offered to patients with hypogonadism in different forms based on the presence or not of gynecomastia. Subjects with hypogonadism and gynecomastia were treated with 2.5% transdermal dihydrotestosterone (DHT) gel, a non-aromatizable ART, at the daily dose of 2 applications on each breast (one in the morning and one in the evening). Patients with hypogonadism without gynecomastia were treated with 2% testosterone (T) gel at the daily dose of 50 mg (five puffs/day). We preferred the use of gel to avoid any additional liver load in subjects already under the liver-affecting mitotane treatment, and because the gel manageability and rapid suspension in case of side effects.

LH, SHBG, TT, and cFT were assessed in all patients at baseline (T0) and 6 (T1), 12 (T2) and 18 (T3) months after AMT start (see below). In addition, TT, SHBG, cFT, and LH and sexual function were evaluated in eight and six men, respectively, before (T3) and six months after (T4) ART (see below).

### Biochemical evaluation

Biochemical evaluation was performed in single reference laboratory, the General Laboratory of Careggi Hospital. The Laboratory is part of AOUC (Azienda Ospedaliero-Universitaria Careggi), is certificated by the Tuscany region, and works according to ISO 9001:2015. Internal quality controls (IQC) are periodically reviewed by the direction of the Laboratory and External Quality Assessment (EQA) schemes are provided and evaluated by third part institution QualiMedLab (CNR, Pisa) and Reference Regional Centre for EQA (CRRVEQ).

Blood samples were drawn in the morning, after an overnight fast, and were immediately centrifuged at 3.000 rpm for 20 minutes. In particular, at T4 (after six months of ART), blood samples were drawn two hours after the application of T or DHT gel (see above) according to the Endocrine Society guidelines (25). LH, TT and SHBG were measured by electrochemiluminescence (ECLIA) method on COBAS 6000 (Roche Diagnostic, Mannheim, Germany). In particular, TT was evaluated by the electrochemiluminescence immunoassay “ECLIA”, intended for use on Elecsys Testosterone II and cobas e immunoassay analyzers; for details see https://labogids.sintmaria.be/sites/default/files/files/testosteron_ii_2017-11_v9.pdf) standardized via ID−GC/MS (“Isotope Dilution - Gas Chromatography/Mass Spectrometry”) (26, 27), which shows good agreement with mass spectrometry in the distribution of results for TT (28). To ensure day-to-day consistency of an analytical process, two levels Bio-Rad internal IQC samples were processed before and after the sample measurements. An External Quality Assurance (EQA) systems to evaluate accuracy and bias was adopted by laboratory using QualiMedLab (CNR, Pisa) EQA controls. A one-year EQA schemes coefficients of variation (CVs) for these methods were 6.0%, 9.0% and 5.6% for LH, TT and SHBG, respectively. EQA sample at cut-off concentration (300 ng/dL) showed a CV and u% of 4.3% and 0.48%, respectively. LH, TT and SHBG assay sensitivity are 0.2 IU/L, 0.42 nmol/l and 0.35 nmol/l, respectively, with a Limit of Quantitation of TT of 0.416 nmoli/l. Calculated free testosterone (cFT) was derived according to the Vermeulen’s formula (available at http://www.issam.ch/freetesto.htm).

Since patients under AMT show a relevant increase in SHBG levels (19–22), in our study, hypogonadism was diagnosed using cFT instead of TT, to increase the specificity of T deficiency detection (low cFT levels) even in men with apparent normal TT levels. A cFT < 225 pmol/l was considered to define hypogonadism (29), while a LH cut-off of 9.4 U/l was used to distinguish between hypo- and hyper-gonadotropic hypogonadism. According to the largely accepted definition derived from the European Male Ageing Study (30), LH < 9.4 U/l defines “secondary or hypogonadotropic hypogonadism”, while LH ≥ 9.4 U/l defines “hypergonadotropic hypogonadism”. In addition, the contemporary presence of LH ≥ 9.4 U/l and cFT ≥ 225 pmol/L defines “compensated hypogonadism” (31). Finally, the presence of hypo- and/or hyper-gonadotropic hypogonadism was defined as “overt hypogonadism”, excluding eugonadism and/or “compensated” hypogonadism.

Plasma mitotane concentration was measured by the Lysosafe service provided by HRA Pharma (http://www.lysosafe.com) at Cochin Hospital Laboratory (Paris, France). Quantification of mitotane plasma concentrations was performed using a validated liquid chromatography method coupled with UV-detection. Mitotane and its internal standard (p,p’DDE) were extracted using a one-step extraction method (protein precipitation). The calibration was linear in the range 0.5-25 mg/L. The intra and inter-precision for the four internal quality controls (0.7, 4, 12.5 and 20 mg/L) were below 4.4 and 9.7%, respectively; and the intra and inter-accuracy ranged from 92.8 to 109.7%. The accuracy of the method was also ensured by the participation to an external quality assessment Scheme including five French Hospital Laboratories.

### Evaluation of sexual function

A standard question “Do you agree to be investigated about your sexuality?” was raised to all patients. The “yes” responders were asked to complete the International Index of Erectile Function-15 (IIEF-15) (32), in its Italian form. The domains investigated by the IIEF-15 are: erectile function, orgasmic function, sexual desire, intercourse satisfaction and overall satisfaction. Erectile function was assessed using the IIEF-15-erectile function domain (EFD) (33). According to the IIEF-15-EFD score, the severity of erectile dysfunction (ED) can be categorized as: no ED (score 30-26), mild ED (score 25-22), mild-to-moderate ED (score 21-17), moderate ED (score 16-11) and severe ED (score 10-6) (33). Since scores between 25 and 22 suggest “very mild” ED, it is not likely to be a major problem for men in engaging coitus, whereas threshold of 22, identifying a more severe problem, can be considered as indicative of a more relevant clinical ED (34). Hence, in our study, ED was defined for an IIEF-15-EFD score < 22.

### Statistical analysis

To summarize patients’ clinical characteristics, we used descriptive statistics. Continuous data were expressed as mean ± standard deviation (SD) when normally distributed, or as medians (quartiles) for non-normal distribution, while categorical data were indicated as percentages, unless otherwise specified. Correlations were assessed using Spearman’s or Pearson’s method, whenever appropriate. One-way ANOVA test for repeated measures was used to compare more than two groups, i.e. hormonal (TT, SHBG, cFT and LH) levels at baseline (T0), T1, T2 and T3. The paired two-sided Student’s *t-*test was used to compare hormonal (LH, SHBG, TT and cFT) levels in two groups and IIEF-15 total and subdomains scores at T3 and T4.

Statistical analysis was performed with SPSS (SPSS, Inc., Chicago, IL, USA) for Windows 27, GraphPad Prism version 9.0.0 for Windows, GraphPad Software, San Diego, California USA, http://www.graphpad.com, and Origin version 6.1 for Windows.

A p-value < 0.05 was considered significant.

All the figures were prepared using GraphPad Prism version 9.0.0 for Windows, GraphPad Software, San Diego, California USA, http://www.graphpad.com and Origin version 6.1 for Windows.

## Results

### Clinical and hormonal characteristics of the entire patients’ cohort at baseline

Patients surgically treated for ACC who underwent AMT (n=10) had a mean age of 43.0 ± 14.2 years. The main clinical and hormonal baseline characteristics of the patients, as well as the main ACC features, are summarized in **Table 1**. At baseline, secondary hypogonadism and gynecomastia were detected in three patients (30%) (**Table 1**), while no patient complained of sexual dysfunction.

**Table 1:** Main clinical, hormonal and oncological features of the sample at baseline.

			N = 10
	%	Mean values	Median values
Clinical parameters			
**Age (years)**		43.0 ± 14.2	47.0 [29-51.75]
**Current smokers (%)**	0.0%		
**Past smokers (%)**	0.0%		
**Daily alcohol consumption (%)**	0.0%		
**Body mass index (kg/m^2^)**		27.8 ± 4.5	28.2 [23.4-33.3]
**Waistline (cm)**		110.9 ± 11.6	102.0 [89.8-115.1]
**Systolic blood pressure (mmHg)**		131.0 ± 19.0	132.5 [117.5-140.0]
**Diastolic blood pressure (mmHg)**		81.5 ± 12.7	85.0 [75.0-90.0]
**Mean testis volume (Prader) (ml)**		13.6 ± 2.0	14.0 [12.0-15.2]
**Gynecomastia (%)**	30%		
**Hypogonadism^a^ (%)**	30%		
**Current medications^b^ (%)**			
- **Hydrocortisone/ cortisone acetate**	100%		
- **Fludrocortisone**	20%		
- **Levothyroxine**	70%		
- **Lipid-lowering therapy**	50%		
			
Hormonal parameters			
**LH (U/l)**		3.3 ± 1.8	4.0 [3.3-4.4]
**Total testosterone (nmol/l)**		14.6 ± 4.8	14.6 [10.5-18.9]
**Sex hormone binding globulin (nmol/l)**		36.6 ± 13.2	33.3 [27.2-43.7]
**Calculated free testosterone (pmol/l)**		296.4 ± 135.4	271.5 [187.2-364.2]
			
Tumour related parameters			
***Tumour size (cm)***		10.8 ± 5.2	9.2 [4.3-15.5]
***ENSAT tumour stage at the start of AMT (%)*** **Stage I** **Stage II** **Stage III** **Stage IV**	20% 50% 20% 10%		
***Hormonal secretion (%)*** **Cortisol** **Non functional tumors** **Cortisol and androgens** **Androgens**	50% 30% 10% 10%		
***Weiss score (%)*** **4** **5** **6** **7** **8**	10% 10% 20% 20% 40%		

a All the hypogonadal patients showed secondary hypogonadism at baseline.

b Supportive/replacement treatments. AMT, adjuvant mitotane therapy.

### Adjuvant mitotane therapy (AMT)

**Table 2** shows the mitotane minimum and maximum daily dose, the mean and maximum levels reached and the TTR in each patient and in the entire cohort. The mitotane plasma mean concentration during AMT was 12.5 ± 2.6 mg/L. The mean TTR in the whole sample was 14.2 ± 13.4 months. Out of ten patients, two never reached the target range.

**Table 2:** Mitotane daily dose, mitotane levels and time in target range (TTR).

Patients	Minimum mitotane daily dose	Maximum mitotane daily dose	Mitotane maximum levels	Mitotane mean levels	TTR
	mg/day	mg/day	mg/L	mg/L	months
**1**	2000	3000	13.60	10.22	0
**2**	2000	4500	24.33	11.15	5.5
**3**	1500	2500	26.76	14.89	42.2
**4**	2000	3000	18.80	13.47	16.4
**5**	2000	3000	17.12	7.39	0
**6**	1500	2500	25.16	14.44	5.2
**7**	3000	3000	13.60	10.53	0
**8**	2000	4000	18.50	13.20	23.7
**9**	2000	3000	18.80	13.47	18.3
**10**	1000	3000	32.00	16.05	33.3
**Overall**	1900 ± 516.4	3150 ± 625.8	20.7 ± 6.3	12.5 ± 2.6	14.2 ±13.4

### LH, SHBG, TT, cFT levels and hypogonadal status during AMT

**Figure 1:**
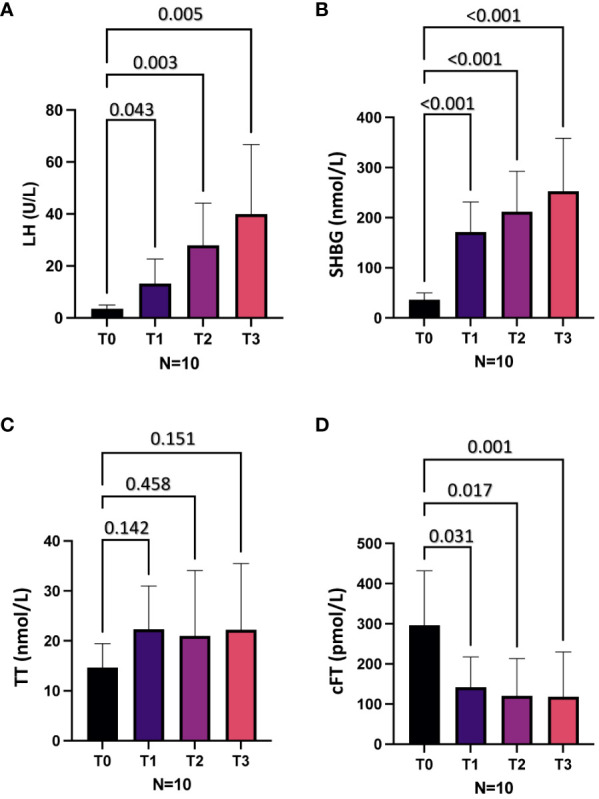
Hormonal parameters at baseline (T0) and after 6 (T1), 12 (T2) and 18 (T3) months of mitotane therapy in the whole cohort. **(A)**, Luteinizing hormone (LH) levels. **(B)**, Sex hormone binding globulin (SHBG) levels. **(C)**, Total testosterone (TT) levels. **(D)**, Calculated free testosterone (cFT) levels.

**Figure 1** shows the mean levels of LH, SHBG, TT and cFT at baseline (T0) and 6 (T1), 12 (T2) and 18 (T3) months after the initiation of AMT in all patients. Compared to baseline, at all the follow-up time points we observed significantly higher levels of LH and SHBG, which showed a progressive increase, and lower cFT levels (all p < 0.05; **Figures 1A, B, D**, respectively), while no significant change in TT levels were observed (**Figure 1C**). Of note, no significant difference in physical examination-related parameters, including body mass index and waistline, was observed in each patient at different time points (not shown).

**Figure 2:**
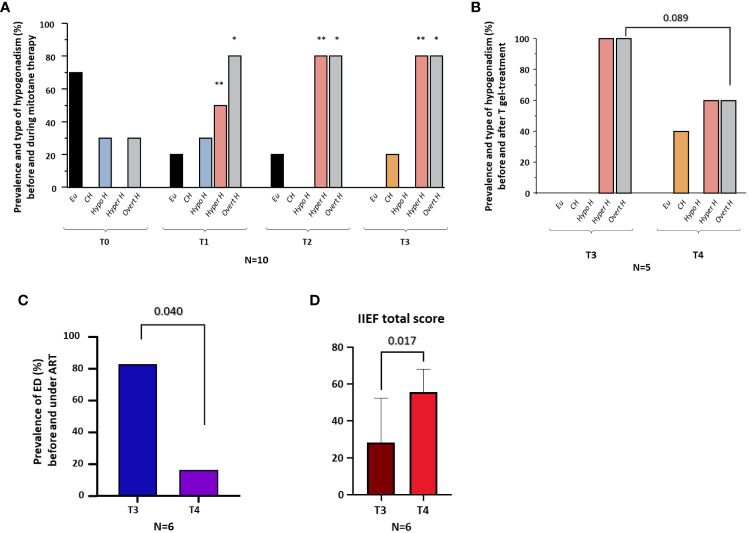
Prevalence and type of hypogonadism before (T0) and during (T1-T3) mitotane therapy; prevalence and type of hypogonadism before (T3) and six months after (T4) T gel therapy; prevalence of erectile dysfunction (ED) before (T3) and six months after (T4) androgen replacement therapy (ART); IIEF total score. **(A)**, Prevalence of eugonadism (Eu), compensated hypogonadism (CH), hypogonadotropic hypogonadism (Hypo H), hypergonadotropic hypogonadism (Hyper H) and overt hypogonadism (Overt H) in the whole cohort at baseline (T0) and 6 (T1), 12 (T2) and 18 (T3) months after the initiation of mitotane therapy. **(B)**, Prevalence of eugonadism (Eu), compensated hypogonadism (CH), hypogonadotropic hypogonadism (Hypo H), hypergonadotropic hypogonadism (Hyper H), and overt hypogonadism (Overt H) in five men before (T3) and after six months (T4) of T gel therapy. **(C)**, Prevalence of erectile dysfunction (ED) in six men before (T3) and after six months (T4) of androgen replacement therapy (ART, including patients under T gel [n=3] and DHT gel [n=3] therapy). **(D)**, Total score of International Index of Erectile Function-15 (IIEF-15) in six men before (T3) and after six months (T4) of ART. *p <0.05, comparing the prevalence of overt hypogonadism at T1, T2 and T3 *vs.* T0. **p <0.05, comparing the prevalence of hypergonadotropic hypogonadism at T1, T2 and T3 *vs.* T0.

On the basis of cFT and LH levels at T0, three patients (30%) showed hypogonadotropic hypogonadism and seven (70%) eugonadism. At T1, five patients (50%) showed hypergonadotropic hypogonadism, three (30%) hypogonadotropic hypogonadism and two (20%) eugonadism. At T2, eight patients (80%) showed hypergonadotropic hypogonadism and two (20%) eugonadism. At T3, eight patients (80%) showed hypergonadotropic hypogonadism and two (20%) compensated hypogonadism. Compared to T0, the prevalence of overt hypogonadism, as well as of hypergonadotropic hypogonadism, was higher at all-time points (T1, T2, T3) after AMT initiation (all p < 0.05) (**Figure 2A**).

Interestingly, we found no associations between AMT-related parameters (minimum and maximum mitotane daily doses, mean and maximum mitotane levels and TTR) and hormonal (TT, cFT, SHBG and LH) levels at any time (T1, T2, T3) (not shown).

### Evaluation of LH, SHBG, TT, and cFT levels and sexual function before and after ART

**Figure 3:**
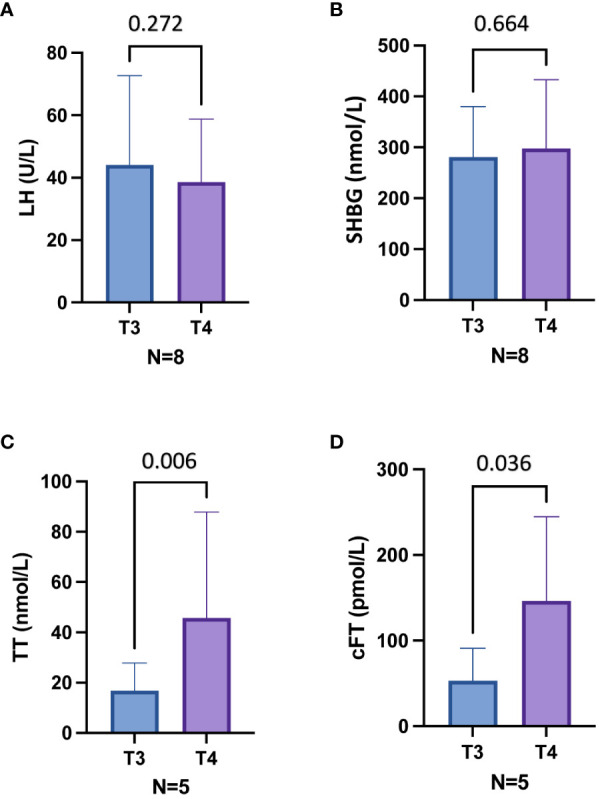
Hormonal parameters before (T3) and six months after (T4) androgen replacement therapy (ART) in eight patients with overt hypogonadism **(A, B)** and in five patients treated with T gel **(C, D)**. Out of eight patients, five have been treated with T gel and three with DHT gel. **(A)**, Luteinizing hormone (LH) levels in eight patients treated with ART. **(B)**, Sex hormone binding globulin (SHBG) levels in eight patients treated with ART. **(C)**, Total testosterone (TT) levels in five patients treated with T gel. **(D)**, Calculated free testosterone (cFT) levels in five patients treated with T gel. T3, 18 months after mitotane therapy; T4, 24 months after mitotane therapy and treated with ART since the last 6 months.

At T3, ART was offered to subjects with overt hypogonadism (n=8) (**Table 3**). In particular, three patients with overt hypogonadism and gynecomastia (cases #3, #4, #5) underwent treatment with DHT gel, while five patients with overt hypogonadism and without gynecomastia (cases #1, #6, #8, #9, #10) underwent treatment with T gel (**Table 3**; see Methods section). Comparing hormonal levels before (T3) and after six months (T4) of ART, no significant differences in LH and SHBG levels were observed (**Figures 3A, B**). Conversely, in the five T-gel-treated patients, after six months of T replacement therapy a significant increase in cFT levels was observed (**Figure 3D**), as well as a trend toward a significant increase in TT levels (**Figure 3C**). Obviously, in those patients under DHT-treatment, TT and cFT levels were not considered. In addition, out of the five patients who showed overt hypogonadism at T3, and therefore started T gel-treatment, two switched to compensated hypogonadism at T4, with a statistical trend (p = 0.089) toward the reduction of overt hypogonadism frequency (**Figure 2B**).

**Table 3:** Presence of overt hypogonadism and gynecomastia after 18 months of mitotane therapy (T3), patients treated with androgen replacement therapy (ART) and type of ART, and patients who agreed to be investigated about their sexuality.

Patients	Gynecomastia	Overt hypogonadism	Androgen replacement therapy (ART)	Patients who agreed to be investigated about sexuality
**1**	No	Yes	T gel 2 %	Yes
**2**	No	No	–	No
**3**	Yes	Yes	DHT gel 2.5%	Yes
**4**	Yes	Yes	DHT gel 2.5%	Yes
**5**	Yes	Yes	DHT gel 2.5%	Yes
**6**	No	Yes	T gel 2 %	Yes
**7**	No	No	–	No
**8**	No	Yes	T gel 2%	Yes
**9**	No	Yes	T gel 2%	No
**10**	No	Yes	T gel 2%	No

T, Testosterone; DHT, Dihydrotestosterone.

In the six patients from whom sexuality was investigated, sexual function data were available starting from T3 (see **Supplementary Table 1**). At T3, five out of six patients (83.3%) had ED (IIEF-15-EFD score < 22) (**Figure 2C**). All the six patients started ART, three (#3, #4, #5) with DHT gel and three (#1, #6, #8) with T gel (**Table 3**). At T4, after six months of ART, we observed a significant decrease in ED prevalence (83.3% at T3 *vs.* 16.7% at T4, p = 0.040) (**Figure 2C**) associated with a significant increase in the all IIEF-15 subdomains and total score (**Figures 2D**, **Supplementary Figures 1A-E**). **Supplementary Table 1** reports the starting (at T3) and the final (at T4) values of the IIEF-15 total and subdomains scores for each patient.

## Discussion

### Main findings of the study

In this study LH, SHBG, TT and cFT variations as well as the prevalence and type of hypogonadism before and after AMT were evaluated in ten surgically treated ACC male patients under AMT. In addition, we evaluated the changes in LH, SHBG, TT, and cFT levels after six months of ART administered in patients who showed overt hypogonadism (n=8). In six patients who had agreed to be investigated about their sexuality, we further investigated the sexual function before and after six months of ART by using the IIEF-15 questionnaire. Essentially, we found that during AMT, patients showed lower cFT levels as well as higher SHBG and LH levels compared to baseline, without significant variations in TT levels over time, most probably because of SHBG increase. At 12 and 18 months-treatment with AMT, 80% of the patients showed overt hypergonadotropic hypogonadism. ART treatment with T gel of the hypogonadal patients subgroup resulted in a significant increase in cFT levels. When evaluating ED in AMT-treated patients, its prevalence (IIEF-15-EFD-defined) was 83.3% eighteen months of treatment. In addition, in six months ART (T or DHT gel)-treated hypogonadal patients, we observed a significant improvement in IIEF-15 total and subdomains scores, (including the EFD score), with an overall significant decrease in ED prevalence to 16.7%.

### Adjuvant mitotane therapy (AMT) and LH, SHBG, TT, and cFT levels changes over time

In the present study, we reported LH, SHBG, TT, and cFT levels changes over time during AMT. In particular, we found that during AMT, patients showed lower cFT levels as well as higher SHBG and LH levels compared to baseline, without significant variations in TT levels over time. It has been demonstrated that mitotane causes a rise in the hepatic SHBG synthesis and release (18) that can lead to a reduction in cFT levels, despite frequent normal or elevated TT levels, and a compensatory elevation of LH. The increase in LH can, in turn, compensate the cFT reduction in some cases, leading to compensated hypogonadism. Since physical examination-related parameters, including body mass index and waistline, did not change in each patient at different time points, SHBG increase, and related cFT reduction, can be mainly attributed to AMT, excluding possible variations of SHBG and testosterone levels related to changes in fat mass, being SHBG and testosterone inversely associated with body mass index (35). The hormonal status in ACC patients under AMT has been evaluated previously only in four studies (17–20), with partially discordant results. A first study (17) reported a significant surge of sex hormone binding globulin (SHBG) levels associated with an initial rise in total testosterone (TT) levels, followed by a reduction, and with a decrease in calculated free testosterone (cFT) levels, with no changes in gonadotropins levels. A second study (18) reported a significant increase in SHBG, a decrease in cFT, but no change in TT levels and gonadotropins. A third study (19) reported an increase in TT but not in cFT levels, along with an increased level of luteinizing hormone (LH). A most recent study reported a cumulative prevalence of hypogonadism of 87.5%, however LH levels were not assessed and the type of hypogonadism was not evaluated (20). Of note, the difference in results reported in previous studies might in part be due to differences in mitotane regimen, timing of blood sampling and laboratory methods for mitotane measurement, as well as in the circulating levels of the drug.

### Adjuvant mitotane therapy (AMT) and prevalence and type of hypogonadism

In the present study, we reported a high prevalence of overt hypogonadism in AMT male patients, timely associated with AMT duration, reaching 80% of cases after twelve and eighteen months of AMT. The time dependent SHBG and LH increase associated with lower cFT levels with no significant changes in TT levels observed in our patients might explain the higher prevalence of hypogonadism detected respect to a previous study, reporting hypogonadism in about one out of three patients under mitotane therapy (16). Of note, patients with overt hypogonadism showed a hypergonadotropic form.

At baseline, we observed that 30% of patients already had hypogonadism but with a hypogonadotropic form. This form of hypogonadism could be due either to the excess of cortisol if present, and/or to the tumor-related cachectic condition (24). After twelve and eighteen months of AMT, 80% of patients showed hypergonadotropic hypogonadism, and, at eighteen months, the remaining 20% showed compensated hypogonadism (high LH with normal cFT levels). The induction of hypergonadotropic hypogonadism was timely associated with AMT duration. However, no association was found between the onset of hypogonadism and mitotane levels or with TTR, in agreement with a previous study (20). Differently, other authors reported an inverse correlation between mitotane and TT levels (22). The present report of hypergonadotropic hypogonadism in surgically treated ACC patients under AMT is a new finding. In fact, two previous studies reported the onset of hypogonadotropic hypogonadism in patients treated with mitotane (19, 20), while one study found an increase in LH values ​​without the development of overt hypogonadism (21). As reported above, the differences between the results of these studies could be in part related to the different mitotane concentration reached, time of evaluation and the laboratory methods used.

In our study, the number of patients that reached a mitotane level higher than 14 mg/l is in line with the literature data (36). As reported above, mitotane causes a rise in SHBG levels (18) that can lead to a reduction in cFT levels and a compensatory elevation of LH. The increase in LH can, in turn, compensate the cFT reduction. The compensatory elevation of LH levels can explain the two cases of compensated hypogonadism observed in our study, while a direct testicular damage of mitotane might explain the lack of compensation and the induction of a hypergonadotropic hypogonadism observed in most of the cases. Accordingly, a previous case report (37) showed that a patient affected by a cortisol-secreting ACC treated with mitotane developed hypergonadotropic hypogonadism associated with ED (37). The patient underwent a testicular biopsy which demonstrated a mitotane-related direct testicular toxicity. This is not surprising because mitotane is toxic for the steroid-producing cells, present not only in the adrenal gland but also as Leydig cells in the testis. The causative role of AMT in the aforementioned alterations was supported by the gradual improvement of libido observed in the patient after therapy discontinuation (37). Regarding this case report (37), two different aspects should be underlined. First, in the presence of hypercortisolism in a patient affected by a cortisol-secreting ACC, a hypogonatropic hypogonadism is expected, while the high levels of gonadotropins observed in that patient strengthen the hypothesis of a crucial negative effect of mitotane at testicular level. Second, the patient was treated with a very high dose of mitotane (6000 mg every 12 hours) which was interrupted due to gastrointestinal and central nervous system side effects (37). Hence, further investigations at the testicular level are needed in patients treated with mitotane in an adjuvant setting.

### Androgen replacement therapy (ART) and LH, SHBG, TT, and cFT levels changes

Considering ART, in the subset of hypogonadal men treated with T gel we observed a significant increase in cFT levels and a trend toward an increase in TT levels after six months of treatment, and a shift of two out of five patients from hypergonadotropic to compensated hypogonadism. Conversely, no significant differences in LH and SHBG levels were observed in all patients treated with ART before and after therapy. Our findings, if supported by future studies, could suggest the use of higher doses of testosterone than those currently used foe the replacement therapy (TRT) in mitotane-induced hypogonadism. A limitation of our study is that we have not measured the DHT levels but is possible that the above considerations for TRT should be extended also to the DHT therapy.

### Sexual function of ACC patients under AMT before and after ART

Regarding sexual function, six patients agreed to be investigated after eighteen months from the initiation of AMT using the IIEF-15 questionnaire. Interestingly, among them, 83.3% of the patients complained of clinically significant ED, as derived from the IIEF-15-EFD score. Upon ART, a significant reduction to 16.7% in ED prevalence was observed along with an overall improvement of sexual function. It could be hypothesized that the increased cFT levels, observed in men under T gel treatment, and/or the increased androgen receptor stimulation by T or DHT are responsible for the amelioration of sexual functions in the treated patients.

A previous study reported no significant clinical effects of TRT in surgically treated ACC patients under AMT (21), while another study observed an improvement in strength, mood and sexual desire in four out of seven treated patients (19). Larger studies comparing treated and non-treated patients as well as treatment with T or DHT gel are necessary to evaluate the real role of ART therapy in ACC patients treated with mitotane. Furthermore, the evaluation of bone assessment could clarify the role of TRT therapy in this setting.

### Limitations and strengths of the study

This study has some limitations, including the retrospective nature and the limited number of patients studied, partly justified by the fact that ACC is a rare cancer. Hence, larger prospective studies are needed to confirm our results. Another limiting point is the use of the electrochemiluminescence method to evaluate TT levels instead of mass spectrometry. However, TT was assessed using the electrochemiluminescence immunoassay “ECLIA” (see above in the Methods section; https://labogids.sintmaria.be/sites/default/files/files/testosteron_ii_2017-11_v9.pdf), standardized via ID−GC/MS (“Isotope Dilution - Gas Chromatography/Mass Spectrometry”) (26, 27), which has previously been demonstrated to show a good agreement with mass spectrometry in the distribution of TT results (28). However, mass spectrometry assays are considered to have the highest specificity and are preferred, but immunoassays are regarded to possess the ability to discriminate between hypogonadism and eugonadism. A further source of heterogeneity is associated to ART: in fact, five out of eight patients received T gel while three received DHT gel, because of symptomatic gynecomastia. In addition, cutaneous absorption of the gel can vary, despite the standardization of the dosage and the time interval between applications.

The present study has also some strengths. In particular, this study evaluated LH, SHBG, TT, and cFT changes at different times as well as, for the first time, sexual function before and after ART. Furthermore, LH, SHBG, TT, and cFT assessment was performed in a single reference laboratory (General laboratory of Careggi Hospital). Finally, this is the first study focused on the andrological assessment of ACC patients treated with mitotane and considering the prevalence and type of hypogonadism as primary aim, and assessing sexual function through a standardized questionnaire as the IIEF-15.

## Conclusions

AMT is associated with a high prevalence of hypergonadotropic hypogonadism in ACC male patients, regardless of mitotane levels and TTR reached. Primary hypogonadism is essentially due to a rise in SHBG and to the absence of LH-related compensatory increase in T synthesis, likely resulting from a mitotane-induced direct damage to the Leydig cells. Patients who underwent surgery for ACC and subsequent AMT showed a high prevalence of sexual dysfunction, that could be ameliorated by ART. Hence, we suggest a periodic LH, SHBG, TT and cFT monitoring during AMT, as well as an assessment of sexual function, to identify those patients with sexual dysfunction who might benefit from ART to, at least partially, improve their quality of life.

## Data availability statement

The raw data supporting the conclusions of this article will be made available by the authors, without undue reservation.

## Ethics statement

Ethical approval was not required for the studies involving humans because we have the approval to collect samples of patients affected by adrenal diseases: Ethics Committee of University Hospital of Florence protocol code 59/11 version 1.3 date 05/04/2019. Informed consent was obtained from all subjects involved in the study. The studies were conducted in accordance with the local legislation and institutional requirements. The human samples used in this study were acquired from primarily isolated as part of your previous study for which ethical approval was obtained. Written informed consent to participate in this study was not required from the participants or the participants’ legal guardians/next of kin in accordance with the national legislation and the institutional requirements.

## Author contributions

LC: Writing – original draft, Writing – review & editing, Conceptualization, Methodology. CS: Data curation, Formal analysis, Methodology, Software, Writing – review & editing. LN: Data curation, Investigation, Writing – review & editing. GDF: Data curation, Investigation, Writing – review & editing. GC: Data curation, Methodology, Writing – review & editing. ER: Data curation, Writing – review & editing. SM: Data curation, Writing – review & editing. TE: Data curation, Writing – review & editing. FC: Data curation, Writing – review & editing. AF: Data curation, Writing – review & editing. LZ: Data curation, Writing – review & editing. AT: Writing – original draft, Writing – review & editing, Data curation. MMan: Supervision, Writing – review & editing. ML: Supervision, Writing – review & editing. MMag: Supervision, Writing – review & editing. FL: Conceptualization, Data curation, Writing – original draft, Writing – review & editing, Methodology.

## Supplementary material

Supplementary Figure 1 Subdomains scores of the International Index of Erectile Function-15 (IIEF-15) before (T3) and after six months (T4) of androgen replacement therapy (ART) in six patients who agreed to be investigated about their sexuality. Out of six patients, three have been treated with T gel and three with DHT gel **(A)**, IIEF-15-erectile function domain (EFD) scores. **(B)**, IIEF-15-intercourse satisfaction domain scores. **(C)**, IIEF-15-orgasmic function domain scores. **(D)**, IIEF-15-sexual desire domain scores. **(E)**, IIEF-15-overall satisfaction domain scores. T3, 18 months after mitotane therapy; T4, 24 months after mitotane therapy and treated with ART since the last 6 months.
