# Supplementary material for: Hypogonadism and sexual function in men affected by adrenocortical carcinoma under mitotane therapy
Source: Front Endocrinol (Lausanne). 2024 Jan 10;14:1320722. doi: 10.3389/fendo.2023.1320722 (PMC10807287; doi:10.3389/fendo.2023.1320722)
Supplement: Supplementary file 1 [file Table_1.docx]

| N=6 | T3 | | | T4 | | | p value^ |
| --- | --- | --- | --- | --- | --- | --- | --- |
|  | **Mean and median values** | |  | **Mean and median values** |  | |  |
| IIEF-15  total score | 24.2 ± 23.7;  16.5 [5.0-44.5] |  | | 55.8 ± 13.3;  56.0 [51.0-65.0] | |  | **0.016** |
| IIEF-15  subdomains scores |  |  | |  | |  | **0.031** |
| -EFD  score | 11.3 ± 11.7;  8.5 [1.0-22.7] |  | | 24.5 ± 6.8:  29.0 [23.0-30.0] | |  | **0.026** |
| ED  (EFD score < 22) | 83.3% |  | | 16.7% | |  | **0.040** |
| -Intercourse satisfaction subdomain score | 3.0 ± 3.9;  2.0 [0.0-5.5] |  | | 9.5 ± 3.3;  9.0 [5.0-11.0] | |  | **0.025** |
| -Orgasmic function  subdomain score | 2.8 ± 3.8;  1.0 [0.0-6.7] |  | | 9.0 ± 1.5  10.0 [9.0-10.0] | |  | **0.014** |
| -Sexual desire  subdomain score | 3.3 ± 1.9;  3.0 [2.7-4.0] |  | | 5.8 ± 2.3;  6.0 [3.0-8.0] | |  | **0.041** |
| -Overall satisfaction  subdomain score | 3.7 ± 3.2;  2.0 [2.0-5.5] |  | | 7.0 ± 3.0;  6.0 [5.0-10.0] | |  | **0.043** |

**Supplementary table 1. International Index of Erectile Function-15 (IIEF-15) total and subdomains scores before (T3) and after six months (T4) of androgen replacement therapy (ART).** Of six patients who completed the questionnaire, three were treated with DHT gel 2.5% and three with T gel 2%. EFD, erectile function domain; ED, erectile dysfunction. ^Similar p values were obtained comparing the median values (not shown).
