# Supplementary figures and images for: Hypogonadism and sexual function in men affected by adrenocortical carcinoma under mitotane therapy
Source: Front Endocrinol (Lausanne). 2024 Jan 10;14:1320722. doi: 10.3389/fendo.2023.1320722 (PMC10807287; doi:10.3389/fendo.2023.1320722)

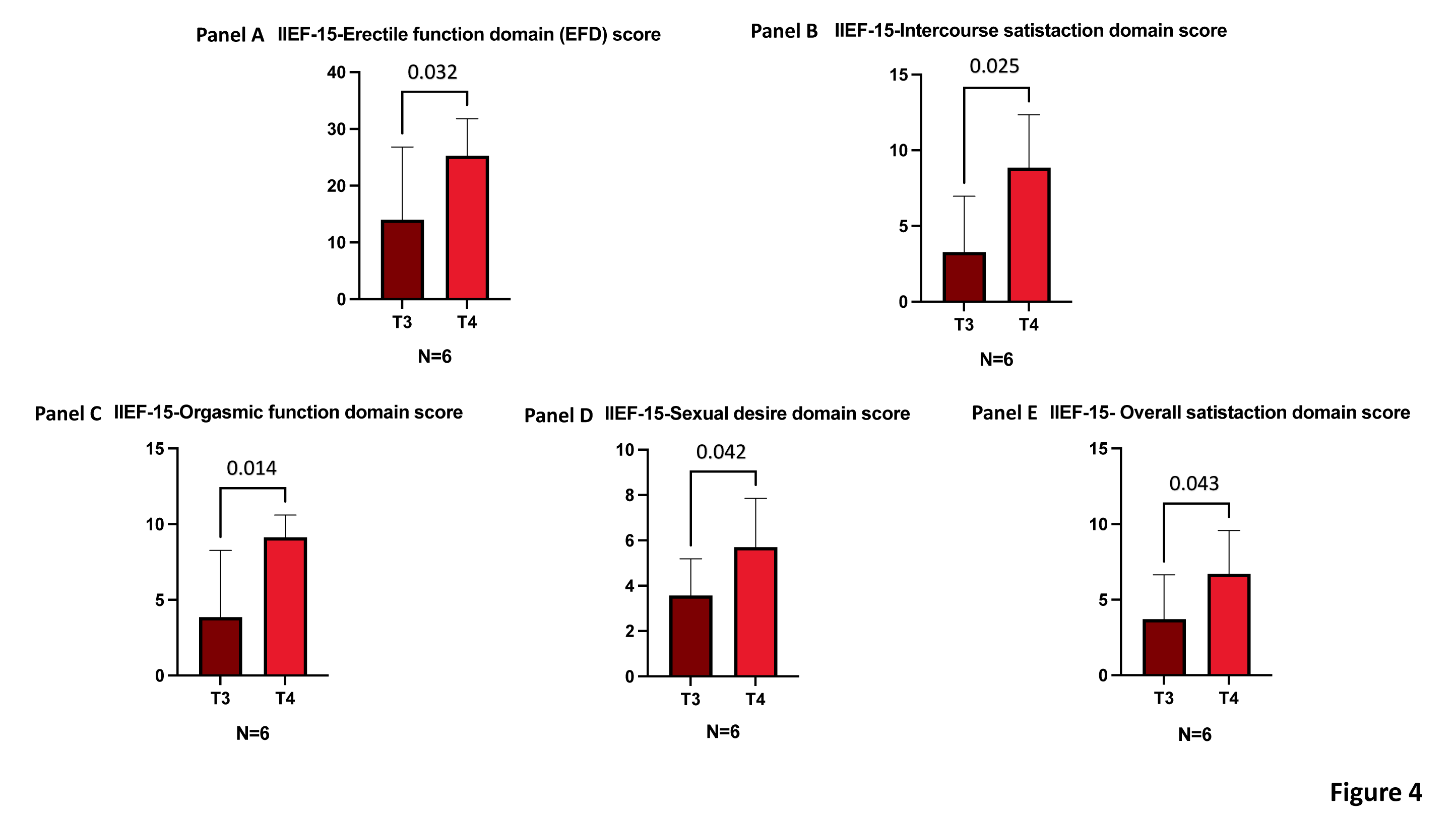

Supplement: Supplementary Figure 1 — Subdomains scores of the International Index of Erectile Function-15 (IIEF-15) before (T3) and after six months (T4) of androgen replacement therapy (ART) in six patients who agreed to be investigated about their sexuality. Out of six patients, three have been treated with T gel and three with DHT gel (A), IIEF-15-erectile function domain (EFD) scores. (B), IIEF-15-intercourse satisfaction domain scores. (C), IIEF-15-orgasmic function domain scores. (D), IIEF-15-sexual desire domain scores. (E), IIEF-15-overall satisfaction domain scores. T3, 18 months after mitotane therapy; T4, 24 months after mitotane therapy and treated with ART since the last 6 months. [file Image_1.tif]
